# Light sheet based volume flow cytometry (VFC) for rapid volume reconstruction and parameter estimation on the go

**DOI:** 10.1038/s41598-021-03902-8

**Published:** 2022-01-07

**Authors:** Prashant Kumar, Prakash Joshi, Jigmi Basumatary, Partha Pratim Mondal

**Affiliations:** Department of Instrumentation and Applied Physics, Indian Institute of Science, Bangalore, 560012 India

**Keywords:** Biotechnology, Health care, Medical research

## Abstract

Optical imaging is paramount for disease diagnosis and to access its progression over time. The proposed optical flow imaging (VFC/iLIFE) is a powerful technique that adds new capabilities (3D volume visualization, organelle-level resolution, and multi-organelle screening) to the existing system. Unlike state-of-the-art point-illumination-based biomedical imaging techniques, the sheet-based VFC technique is capable of single-shot sectional visualization, high throughput interrogation, real-time parameter estimation, and instant volume reconstruction with organelle-level resolution of live specimens. The specimen flow system was realized on a multichannel (Y-type) microfluidic chip that enables visualization of organelle distribution in several cells in-parallel at a relatively high flow-rate (2000 nl/min). The calibration of VFC system requires the study of point emitters (fluorescent beads) at physiologically relevant flow-rates (500–2000 nl/min) for determining flow-induced optical aberration in the system point spread function (PSF). Subsequently, the recorded raw images and volumes were computationally deconvolved with flow-variant PSF to reconstruct the cell volume. High throughput investigation of the mitochondrial network in HeLa cancer cell was carried out at sub-cellular resolution in real-time and critical parameters (mitochondria count and size distribution, morphology, entropy, and cell strain statistics) were determined on-the-go. These parameters determine the physiological state of cells, and the changes over-time, revealing the metastatic progression of diseases. Overall, the developed VFC system enables real-time monitoring of sub-cellular organelle organization at a high-throughput with high-content capacity.

## Introduction

Disease diagnosis and its cure require single platform-based diagnostic systems that are multifunctional, compact, and capable of rapid analysis. Flow based system such as, Flow cytometry (FC) is one such powerful system that is capable of conduct diverse study on a single platform. FCs have shown its potential in a spectrum of fields ranging from optical imaging to biotechnology^1–10^. In short, FC combine the strengths of optical imaging and flow cytometry. Apart from providing the basic functions of conventional cytometry such as count, sort, mix and analyse cells, this also offers metrics that can be used to discern cells based on their morphology and cellular variations^11–21^. FCs facilitate in-depth investigation at cellular level that may have immediate implications in optical physics and biotechnology.

Conventional FCs are constrained by low-throughput (due to sequential cell screening), poor resolution (owing to flow-induced optical aberration and limited photon budget), and complex instrumentation (due to the necessity of sheath fluid-based hydrodynamic focussing)^7,11^. To overcome these limitations, a light sheet-based system is developed. Ever since the first demonstration in 2013^22,23^, light sheet cytometry has brought in paradigm-shift from point to plane based interrogation. In the past few years, light sheet imaging cytometry (LIC) has gained popularity and many research groups have started exploring LIC to incorporate live organism imaging^24^, time-stretch imaging^25^, phase-contrast^26^, high-speed interrogation^27^, fast sectioning^28^, depth-penetration^29^, determination of protein concentration^30^, measurement of intracellular content^18^ and label-free cell identification^31^. Recent techniques that combine light sheet and imaging cytometry include SPIM-fluid help rapid interrogation of zebrafish for massive drug screening^32^, milli-fluidics (capillary) based cytometry that facilitates imaging of phytoplankton^33^, and droplet-based light-sheet cytometry technique for embryo sorting and cell growth assays^34^. These techniques diversify the use of light sheet-based imaging cytometry for screening a broad spectrum of specimens ranging from a single cell to multicellular organisms.

Existing point-based flow cytometry techniques require hydrodynamic focussing of specimen to a narrow space for it to be intersected by the system PSF, while sheet-illumination cross-sections the entire flow channel. This brings-in the advantage of sectional interrogation of a large number of specimens in-parallel. The geometry of light sheet illumination eliminates the complexity associated with hydrodynamic imaging. For detection, widefield based orthogonal detection is employed that can image specimens with diffraction-limited resolution^35–38^. Subsequently, sectional images of the specimen are deconvolved and stacked together in real-time to reconstruct the volume-stack, a feature never reported before. Effectively, the proposed VFC system maps sectional 2D images of the entire specimen onto the 3D space for instant volume reconstruction. VFC is a promising next-generation technology that may find use in biophysics and optical imaging.

**Figure 1:**
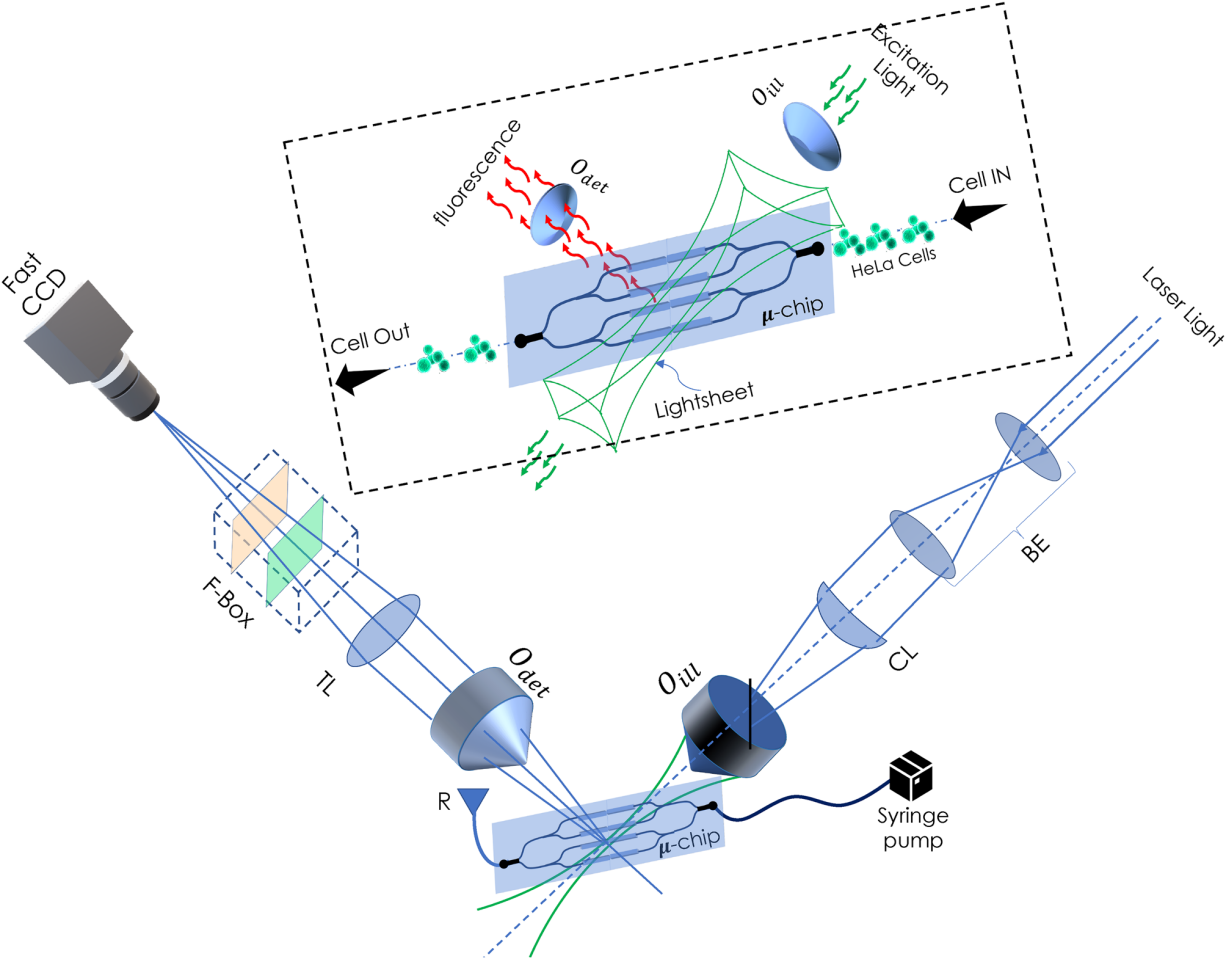
Schematic diagram of the VFC system: Both illumination and detection subsystems are configured in an orthogonal setting for light sheet based interrogation. A laser of 532 nm light is expanded (using beam-expander, BE) and subjected to cylindrical lens ($f=150$ mm)—objective lens (10X, 0.30 NA) combination. This generates diffraction-limited light sheet that sections the entire Y-type 4-microfluidic channel array. Several cells flowing through the channels gets simultaneously interrogated by light sheet. The fluorescence from the labelled mitochondria is collected by detection objective (20X, 0.4 NA), filtered by filter-box (containing a set of longpass and notch filters) and focussed to the CCD camera by tube-lens (TL). The angle between the microfluidic chip and light-sheet illumination is $\approx \,45^{\circ }$. Instant deconvolution is carried out and cell volume is reconstructed. The inset shows an expanded view of light sheet intersecting the microfluidic channel-array.

**Figure 2:**
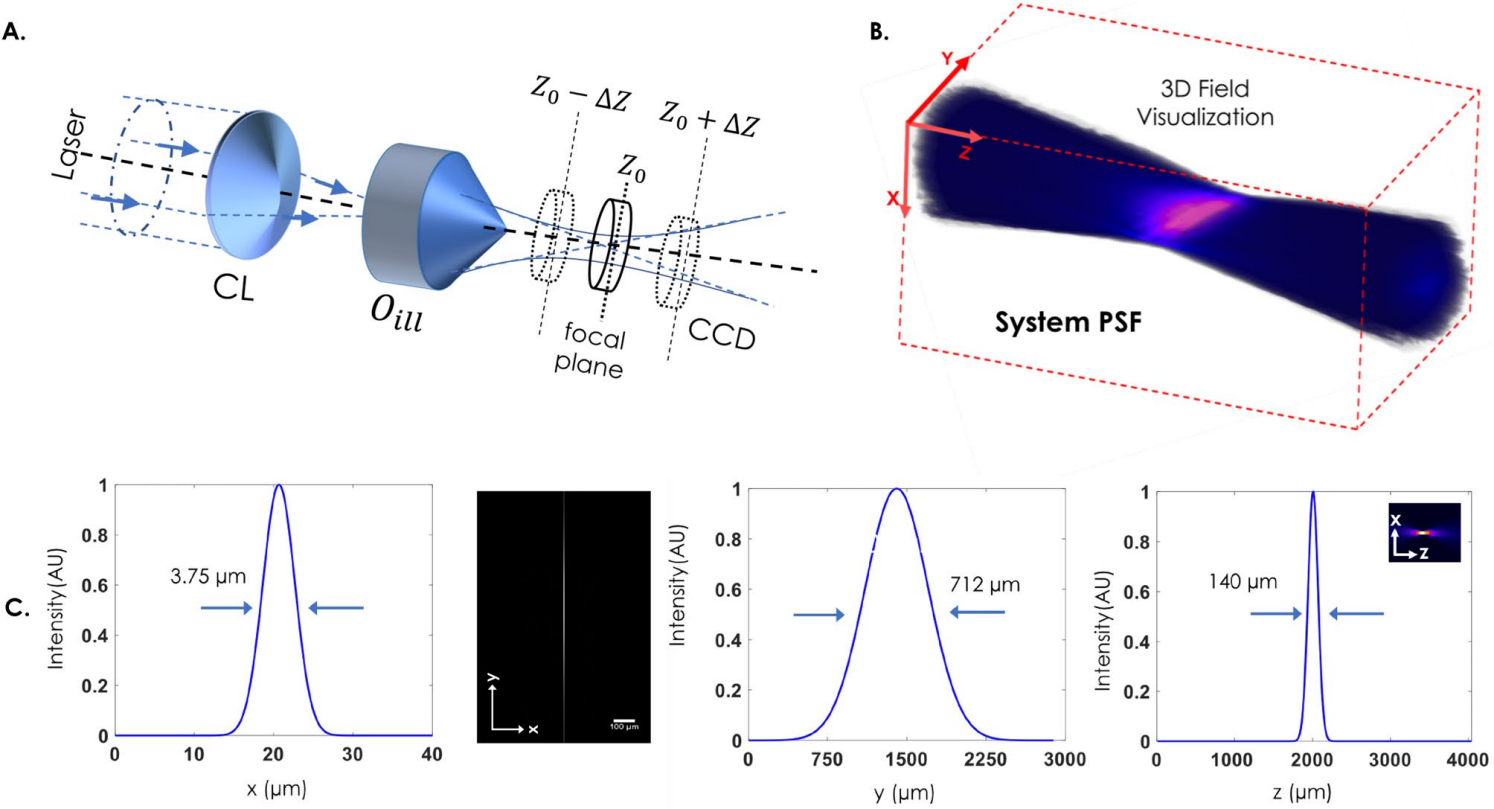
3D lightsheet field visualization: (**A**) Optical set-up to measure the diffraction-limited light sheet field. The CCD camera is placed on a linear-translator and scanned along the optical z-axis to record 2D field intensity. (**B**) The field images are stacked together to reconstruct 3D field. (**C**) XY, XZ and YZ views of three dimensional light sheet field are also shown. The corresponding dimensions of experimentally recorded light sheet shows a light sheet of dimension $\approx 712 \times 140$ µm^2^ along $\,(y\times z)$ and has a thickness of 3.75 µm.

## Results

The development of VFC system is advantageous when compared to traditional systems. The system is capable of determining critical parameters on the go that may pin-point the underlying biological mechanism responsible for disease state and may assist in biomedical diagnosis.

### Light sheet based VFC system

To qualify for a full-fledged biomedical diagnostic health-care system, the traditional cytometry requires new capabilities with diverse functions. Towards this goal, the VFC system is developed to add new functions such as high-resolution cross-sectional imaging, real-time volume reconstruction, critical parameter estimation, and parallel screening of multiple specimens. This technique may further lead to the understanding of cell morphology, intracellular protein concentration, organelle distribution, and their changes from untreated cell type indicating diseased state.

The VFC system consists of three major sub-systems: (1) Light sheet illumination, (2) Microfluidic chip-based cell flow platform, and (3) Orthogonal widefield detection. The illumination sub-system is at the heart of VFC system that uses a sheet-of-light to illuminate the specimen cross-sections as they flow through the cell flow platform. This is unlike the existing state-of-the-art cytometry systems that employs a point-PSF for illumination. In this respect, sheet-based illumination is a paradigm shift that lifts constraints (such as the requirement of hydrodynamic focussing and sequential specimen interrogation) and related complications faced by existing cytometers. The second important aspect of VFC system is the specimen flow system. The existing cytometers require sheath fluid that hydrodynamically focus and collineates the cells to align in the direction of flow. On the contrary, the proposed VFC system employs a microfluidic chip-based cell flow device that does not require the cell to align in a straight line. The light sheet is chosen large enough so that it can cross-section the entire micro-channel array, and in the process optically section specimens flowing through the channels. The details of channel fabrication and microfluidic device is detailed in Supplementary 1 The detection is carried out in an orthogonal widefield mode, which gives the system an unprecedented near diffraction-limited resolution.

Figure 1 shows the schematic diagram of an actual volume flow cytometry system. The system is used in a fluorescence mode for the proposed study related to organelle (mitochondria) imaging. Laser of wavelength 532  nm with a beam-width of 1.5 mm is expanded using a beam-expander to expand the beam by 5 -times. This is essential to fill the back-aperture of cylindrical lens (CL) for utilizing the maximum available aperture angle. The resultant beam is 1D focussed at the back-aperture of illumination objective lens ($O_{ill}$). This gives rise to diffraction-limited light-sheet PSF at the focus of objective lens. The sheet thus generated intersects the near-transparent PDMS-based microfluidic chip. Subsequently, the cells are loaded in the reservoir (R), which is connected to one end of channel array, and the flow is generated by flow-pump attached to the other end. The pump is operated in withdrawal-mode and a continuous flow of cells is ensured at a controlled flow-rate. The mitochondria of cell was labeled with Mitotracker orange ($\lambda _{exc}: \lambda _{emi} = 554\,{\rm nm}: \,576$ nm) dye to visualize the mitochondrial network in a cell as per the developed protocol (see, section F). The fluorescence from flowing cells is collected by the detection objective that is placed orthogonal to the illumination. The light is subsequently focussed by the tube lens (TL, $f=125$  mm) to the fast-CCD camera (with a maximum frame-rate of 2300 full-frame / sec). On its way to the detector, the light is filtered by a combination of filters (bandpass and notch filters) placed in the filter box (F-Box). The inset of Fig. 1 shows the intersection of PDMS microfluidic chip with the light sheet.

**Figure 3:**
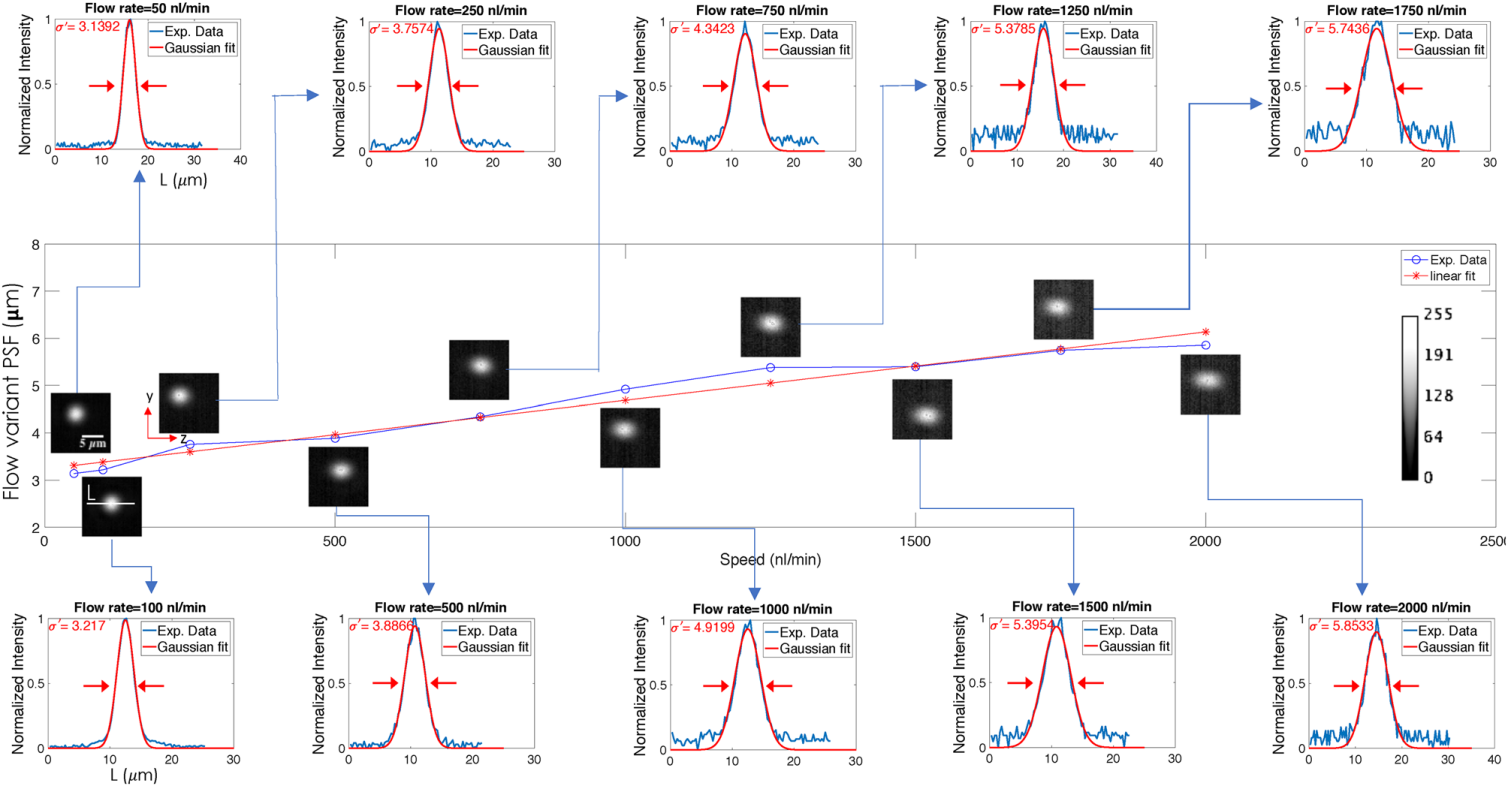
Flow-variant system PSF: The PSF at different flow rates ranging from 50 to 2000 nl/min are shown. Fluorescent beads of size 1 µm is used as point source for determining PSF. With increased flow, the point sources appeared elongated along the flow direction for which the cross-section (across the PSF indicated by white line *L*) is plotted (see, blue curve). To evaluate the apparent elongation caused by motion-blur, the data is fitted with a Gaussian function (see, red line) and the corresponding full-width at half-maximum (FWHM) is calculated. Subsequently, flow-variant PSF size ($\sigma ^\prime = 2\sqrt{2ln2} \sigma _z$) is plotted at different flow rates that indicate a linear increase in apparent size of the system PSF.

**Figure 4:**
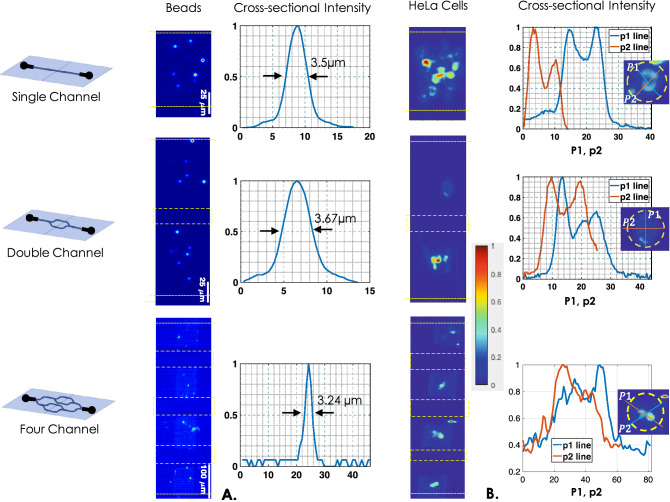
Parallel multichannel visualization: (**A**) Fluorescent beads flowing through different channel types (1, 2 and 4 channels). Beads flowing through the channels are simultaneously illuminated by light sheet and imaged by fast CCD camera. Corresponding intensity plots show FWHM values of beads flowing through the channels. (**B**) Mitochondria-labelled HeLa cells flowing through different channel types and simultaneously imaged in-parallel as they pass through the light sheet, producing 4–8 cross-sectional images. The associated intensity plots show variations indicating mitochondrial network inside the cell.

### Dynamic light sheet characterization

The light field and its interaction with the specimens flowing through PDMS are at the heart of the illumination subsystem. This inherently determines the selectivity and FOV of the imaging system. A focused light sheet reduces the plane thickness, thereby improving cell sectioning ability and boosts the signal-to-background ratio. Figure 2 shows the experimentally measured light field at and around the focus of illumination objective ($O_{ill}$). The field is measured directly by translating a camera (equipped with ND filters) in the beam-path. A CCD camera was placed in the beam-path, and scanned along the optical axis to record the field at each *z*-increment (see, Fig. 2). We have employed step-gradient neutral density (ND) filters to avoid intensity saturation. The scanning was performed by a translator carrying the camera. The 3D recorded field, along with its XY, YZ, and XZ views are shown in Fig. 2. Besides, field is characterization using intensity plots along X, Y and Z axes to determine its size and shape. The shape of light sheet can be assessed from the associated 2D (XY, YZ, and XZ) images. Intensity plots show that the generated light sheet has a dimension of $\approx 712 \times 140$ µm^2^ along $\,(y\times z)$ and a thickness of 3.75 µm (FWHM values). The 3D view displays a planar light sheet field at and around the geometrical focus. Specifically, strong confinement of the field is evident along the z-axis resembling a sheet-like intensity field. The FOV of the system is $\sim 0.09968 \,\mathrm{mm}^2$. The intersection of light sheet field with the cells flowing through the microfluidic channels determine the reconstruction quality of cell volume.

### Flow based dynamic PSF

VFC is a dynamic flow imaging system that requires accurate characterization of system PSF. This has profound implications on image quality. The very fact that a moving target induces blur, distortion (for cells) and optical aberrations compromise system resolution and thereby affect the overall quality of sectional images / cell volume. To determine system PSF during flow, we have used fluorescent beads (of size, $\approx 1$ µm) as point source flowing through the channel at different flow-rates. Figure 3 shows fluorescent beads ($\lambda _{exc}: \lambda _{emi} = 535 \,\mathrm{nm} : \,575$ nm) at flow-rates ranging from 50 to 2000 nl/min. It may be noted that VFC is operated in the mentioned dynamic flow range for the present study. At low flow-rates, the system PSF is largely unperturbed and shows minimal motion-blur. However, the situation changes as the flow-rate is increased to 1000 nl/min, but the blur is visually apparent at around 2000 nl/min. To access the effect of flow, intensity across the bead cross-section is plotted for the entire dynamic range. Subsequently, quantification is carried out by fitting Gaussian, and the apparent size (due to motion-blur) of system PSF size is determined along the flow direction. The beads flowing through the channel are found to be elongated along the flow direction, with its apparent size increased by almost $\approx ( \sigma _{2k\,\mathrm{nl}/\mathrm{min}}/ \sigma _{50\,\mathrm{nl}/\mathrm{min}}) = 1.8645$ fold. This is quite significant considering the fact that we are seeking high resolution at large flow-rates. Throughout the experiment, we have used a frame-rate of 38 frames/sec to collect more photons. Large frame-rates reduce both motion-blur and system resolution (as determined by the number of detected photons). So a balance between flow-rate and camera frames/sec is necessary for quality imaging. The linear relationship between the PSF size and flow-rate is quite evident from the straight-line fit. A small slope of $1.39 \times 10^{-3}$ µm/nl/min ensures that the system operates in linear regime. However, larger flow-rates lead to non-linearity and other effects. All these make us believe that flow in the interval 50–2000 nl/min is linear in nature that leads to proportional changes in the size of system PSF (along the flow direction), and hence appropriate for our study. A complete protocol for data acquisition using the VFC system is discussed in Supplementary 2.

### Simultaneous multichannel visualization of beads and HeLa cells

Real-time volume imaging of multiple cells flowing through the microfluidic channel-array is a daunting task. This paves the way for next-generation flow-based diagnostics. The VFC system is designed to address these issues and overcome the constraints associated with existing state-of-the-art imaging cytometry systems. The key features that distinguish VFC from the existing imaging cytometry system are, (1) multichannel-array based imaging, (2) cross-sectioning ability, (3) organelle-level resolution, (4) high throughput interrogation (due to microchannel array assembly), and (5) volume rendering in real-time. The choice of cylindrical lens - objective lens combination is critical in determining the size of light sheet. We have chosen the combination so that the light sheet sections the specimens flowing through the microchannel array. This ensures parallel interrogation of cells flowing through all the channels, giving it the much-needed advantage of high throughput interrogation. Figure 4 shows fluorescent beads and HeLa cells (fluorescently-labeled) flowing through single, double, and four channels. As a first step, we have flown fluorescent beads to determine dynamic flow parameters such as flow-rate and optimal sample concentration, among others. Experimentally, we observed that the beads are moving fairly in straight line along the flow direction up to a flow-rate of 2500 nL/min. In the steady state condition, bead swings are not observed possibly due to its brief stay in the light-sheet. The corresponding raw data of beads flowing through the microfluidic channel-array is shown in the Supplementary Video 1. We observed that the channels often get blocked at low flow-rates ($< 600 \,\mathrm{nl}/\mathrm{min}$) predominantly due to gravity. Repeated study shows that a high flow rate of 2000 nl/min is appropriate for smooth cell-flow, owing to its large mass (compared to fluorescent beads) and relatively large size (diameter $\sim 15-25$ µm). The study involves freshly prepared cells loaded to the sample reservoir (R in Fig. 1), and flow is induced by the suction-pump ( operated at 2000 nl/min). The corresponding raw data of cells flowing through the channel is shown in the Supplementary Video 2. The cross-sections of the specimens are recorded as they flow through the channel and intersected by the light sheet. The images are then deconvolved using the flow-variant PSF and subsequently stacked together to reconstruct 3D volume. The specimens are flown at 2000 nL/min, and a maximum of 800 cell volumes are recorded per minute. Details of frame-rate, exposure time, and other critical information are discussed in section IV.A. A slow-motion version of a typical HeLa cell as it passes through the light sheet is presented in Supplementary Video 3. The associated plots show internal distribution of mitochondria in the cell flowing through the channel (along white line). The peaks indicate discrete presence of mitochondria / mitochondrial assembly (see intensity plots), and also determines the practical resolution ($\approx 3.5$ µm) of VFC system.

### Cell count statistics

**Figure 5:**
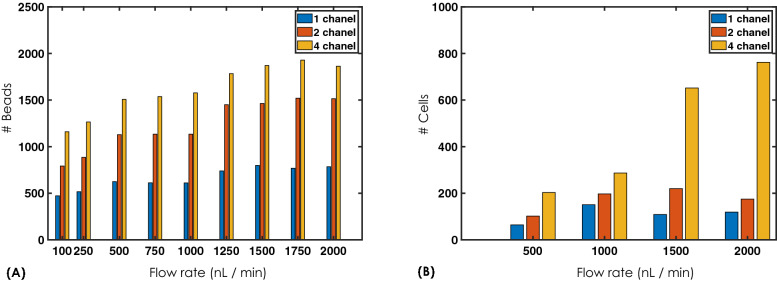
Counting statistics for volume flow cytometry system: (**A**) Fluorescent bead counting for 1, 2 and 4 channels showing a linear behaviour with increasing flow-rates, (**B**) HeLa cell counting shows a rather non-linear behaviour for 1 and 2 channels but displays a linear nature for 4 -channels. This can be attributed to cell-clustering.

The population study of cell ensemble is critical to disease diagnosis and facilitates periodical assessment over time. Proposed VFC has shown promise for high throughput imaging and microfluidic chip-based parallel interrogation of a diverse cell population. Figure 5 shows the count statistics for both the fluorescent beads and HeLa cells at varying flow rates. For beads, we observed a healthy double-fold increment in count statistics for a double channel array while the increment is about 2.5 for a 4-channel array. However, the count for relatively larger specimen (such as HeLa cell) is moderate at low flow-rates ($\approx 200$ per minute at 500 nl/min) and better at large flow-rates ($> 800$ per minute at 2000 nl/min). The variations are observed at intermediate flow-rates. This can be attributed to cell-accumulation near the tube-channel junction and gravity-enforced settling of cells that restrict mobility (owing to their large mass and size). Nonetheless, we noted a healthy cell count of $>800$ for 4- channel array at large flow-rates. Additional constraints associated with flowing HeLa cells are local channel constriction and the affinity of HeLa cells to form clusters. These processes are found to be less prominent at large flow-rates. This is primarily the reason behind relatively large count at high flow-rates (2000 nl/min) compared to low flow-rates (say, 500 nl/min). Our results favor imaging cytometry at large flow-rates.

### Visualization of organelle distribution at large flow

**Figure 6:**
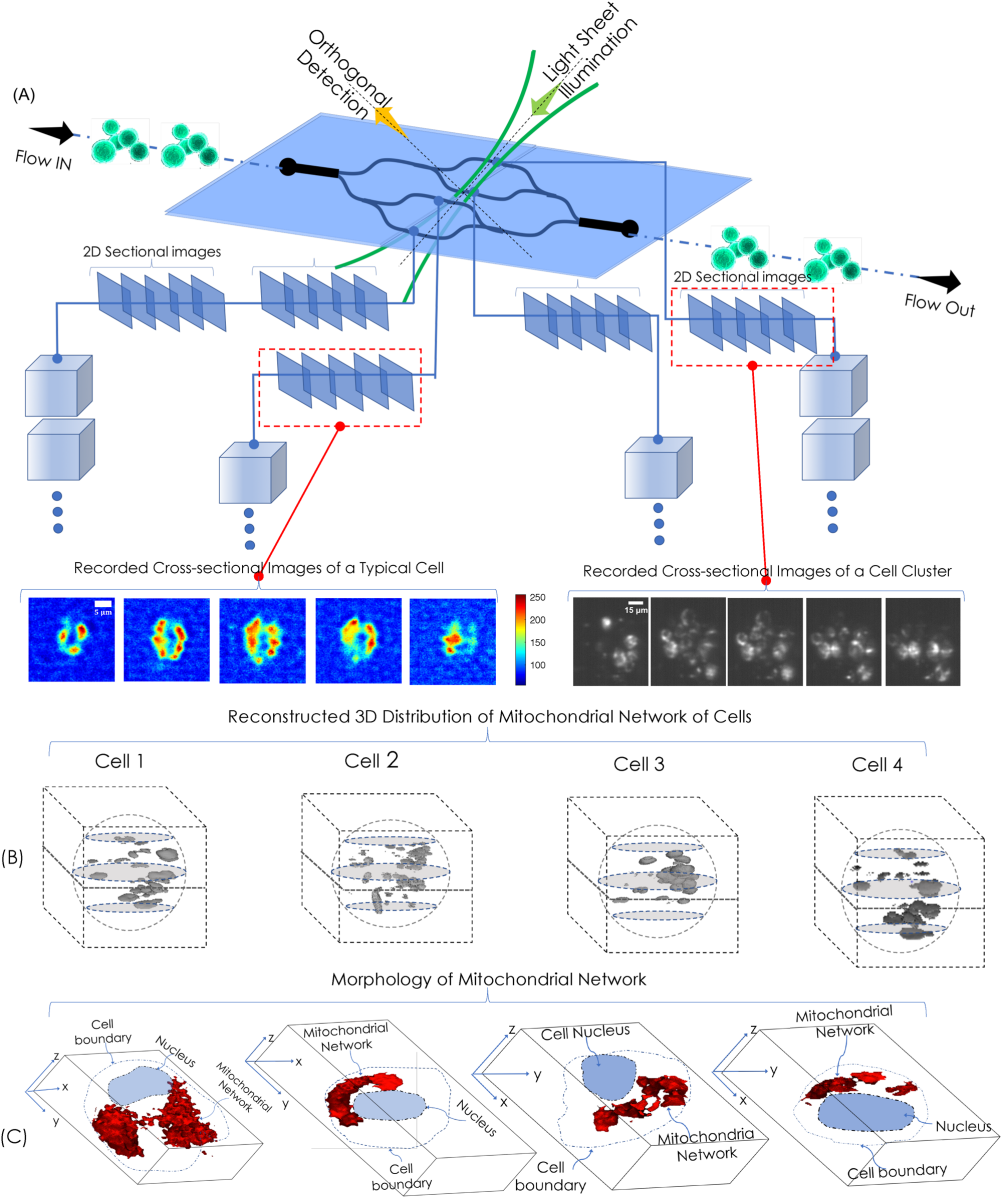
Volume flow cytometry and volume reconstruction: (**A**) A cartoon of VFC data acquisition system beginning from cell interrogation to volume reconstruction on the go. Alongside, a sample of recoded cell and cell-cluster cross-sections are also shown. (**B**) 3D reconstructed cells displaying mitochondria distribution inside flowing Hela cells. (**C**) Reconstructed volume showing mitochondrial network (displayed in red color) inside HeLa cells. For clarity, nucleus and cell boundary are drawn. The reconstruction show organelle-level resolution in flow imaging system.

Visualization of intracellular organelle at high speed and discerning its distribution is a step forward in imaging flow cytometry. The cells interrogated by the sheet illumination provides cross-sectional images of HeLa cells as they pass through the light sheet. Figure 6 shows the process of obtaining sectional images of multiple cells and cell-clusters simultaneously flowing through all the 4 channels. On average, we have observed 3-5 independent single cells per channel at the same time. Using cell preparation protocol and controlling the flow rate, it is possible to ensure that cells do not attach to each other. Occasionally, we have seen cell clusters as well, which is due to the tendency of cells to adhere to one another. VFC involves recording the cross-sections of multiple single cells in parallel, followed by identification and reconstruction. The sectional images are subjected to image reconstruction process (background subtraction and deconvolution) to obtain 3D volume and the distribution of mitochondria in a single HeLa cell (see, Supplementary 3). Schematically the process is shown in Fig. 6 along with the distribution of mitochondrial network for few HeLa cells (see, Fig. 6B) along with its morphology (see, Fig. 6C). The distribution of mitochondria and its morphology are key parameters that indicate general physiological of HeLa cells.

**Figure 7:**
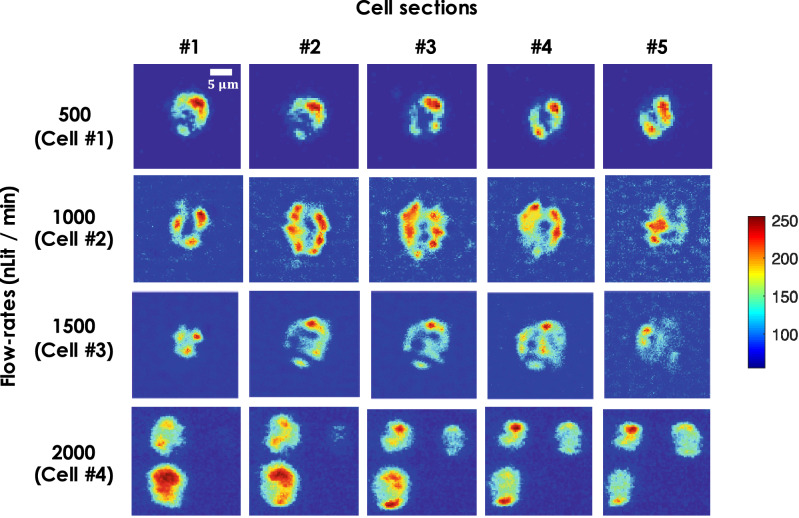
Cell sections: Cross-sectional images of few cells (labelled as, cell $\#1$-$\#4$) flowing through the channel at varying flow-rates, ranging from low (500 nl/min) to high (2000 nl/min) flow-rates.

A few cross-sectional images of mitochondrial network inside a typical HeLa cell as they flow through the light sheet is shown in Fig. 7. The 2D cross-sectional images of mitochondrial distribution inside the cells also shows the dynamic resolution of HSBI system. Better resolved images are evident at low flow-rates (displaying more variations) than the sectional images recorded at high flow-rates. The ability to obtain high resolution images on-the-go add an additional functionality in imaging flow cytometry and opens up future avenues for data analysis. In addition, we have compared VFC with few other state-of-the-art light sheet fluorescence microscopy systems as shown in the Table 1. The performance table gives a qualitative performance and capabilities of VFC when compared to existing techniques.

**Table 1:** Comparative performance between flow-based VFC and the other state-of-the-art light sheet fluorescence microscopy implementations.

	Flow-based VFC	On-chip VFC	Microlens-based VFC
Throughput (cells/min.)	800	$>1000$	100–200
Image quality (Contrast)	High	Moderate	High
Resolution	Organelle-level	Cellular-Level	Organelle-level
Parameter estimation	Count, Area, Strain	–	–

### Biophysical parameter estimation

**Figure 8:**
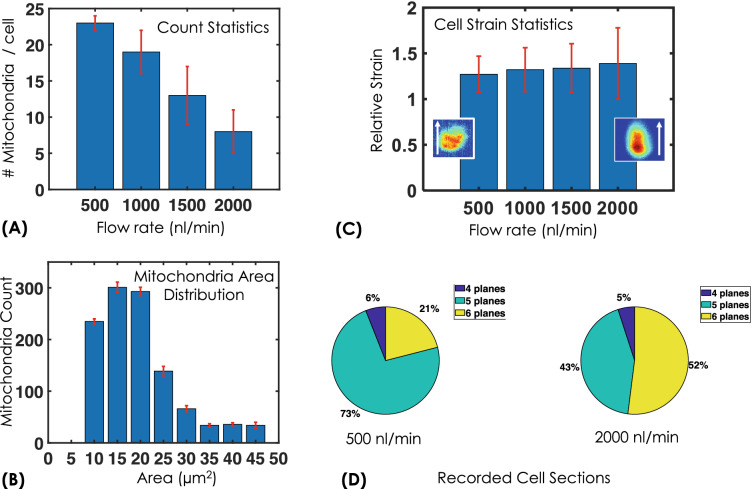
Biophysical parameter estimation: (**A,B,C**) Mitochondria count statistics, size / area distribution and relative cell stress statistics from low (500 nl/min) to high (2000 nl/min) flow-rates. Count statistics show a steady decrease in mitochondria count with increase in flow-rate, whereas mitochondrial area statistics show Gaussian behaviour with its peak at $15$ µm^2^. The corresponding strain statistics indicates linear increase in strain with flow-rate. A typical image of cell under strain is also inserted along with the flow direction (indicated by white arrow) for visualization purpose only. (**D**) Cell strain is also evident in cell-section statistics indicating an increase in the number of cross-sectional planes detected per cell at high flow-rates.

Rapid screening of organelles such as Mitochondria in a large population of cells and real-time estimation of biophysical parameters are critical for cell viability studies. Mitochondria are known to divide / fuse continuously and their size are finely tuned^39,40^. The fission and fusion of mitochondria play key roles in maintaining its integrity. Moreover, distribution of mitochondria contributes to the functioning of organelles that lead to healthy cell growth and its survival. Diseases such as cancer and Alzheimer are known to disturb this balance, causing change in count, its size and distribution of mitochondria in a cell. VFC system has the ability to extract these parameters and related statistics for a large population of cells for organelle-level investigation. Such a system is capable of assisting disease diagnosis at sub-cellular level. To demonstrate the capability of VFC system, we have considered three important parameters (mitochondria count per cell, size distribution and cell strain statistics). Figure 8 shows estimated parameters at varying flow-rates. Figure 8A displays the actual count of mitochondria / clustered-mitochondria per cell. It is evident that the count is high at low flow and decreases monotonically at large flow. This is predictable due to the fact that large flow results in motion-blur and high background that eventually makes it hard to detect small mitochondria. Figure 8B shows that mitochondrial size (area) has a large range from 10–45 $\upmu \,\mathrm{m}^2$ and it peaks at $15 \,\upmu \mathrm{m}^2$ indicate its average size. It is worthwhile to note that area of HeLa cell lies somewhere between 300 and 700  $\upmu \mathrm{m}^2$. In addition, cell strain studies are carried out that indicate the elasticity of cell and its deformability at large flow. After detaching the cells from the petridish surface they assume spherical shape (presumably due to surface tension). When flown the cells elongate along the flow direction and consequently shrinks in the perpendicular direction, assuming the shape of an ellipsoid with *a* and *b* as major and minor axis, respectively. Thus, the metric $S_\sigma \propto (a/b)$ is taken as the measure of flow-induced strain / deformation. Figure 8C shows cell strain from low (500 nl/min) to high (2000 nl/min) flow-rates. A linear increase in strain is evident. In addition, large deformation along flow direction helps in recording more optical sections during high flow ($\sim 2000 \,\mathrm{nl}/\mathrm{min}$) than at low flow (500 nl/min). Associated cell section statistics show that the camera is able to record large number of sections (see, Fig. 8D). The proportion of such cells have jumped to $52\%$ at 2000 nl/min from $21\%$ at 500 nl/min indicating large strain in cell. Another parameter of interest is entropy that determine the nature of distribution (ordered / disordered). This is explained in the subsequent section. To the best of our knowledge, multi-parameter estimation is unknown in imaging flow cytometry, and availability of the biophysical parameters may help understand cellular processes.

## Discussion

The ability to generate high-resolution volume stacks of a large cell population at high throughput has profound applications in biomedical engineering, and medical devices. Data obtained by VFC imaging cytometry contain cellular, multicellular, and intracellular information of a large population, which can be used to classify cell types and access their physiological state (disease / healthy). Moreover, the data can also be used for multiparameter analysis and gather large population statistics. The proposed VFC/iLIFE system may enable cancer cell identification^17,41^, drug screening^10^, and assist biomedical diagnostics^42–44^.

The primary advantage of VFC lies in its ability to reconstruct whole specimen volume at high throughput ($> 800 \,{\rm cells /min}$) with sectional-selectivity and in-principle diffraction-limited spatial resolution. The generation of numerous single-cell volumes in a short period of time has both advantages and disadvantages. The fact that high speed introduces motion-blur, resulting in poor resolution and low speed undermine high-throughput interrogation necessitates a balance between high throughput screening and spatial resolution. Other consequences of low speed are gravity-induced cell accumulation at the bottom of microfluidic channel and the tendency of cells to form clusters. The proposed VFC system mitigates these issues by employing a light sheet based planar interrogation system that can image cells / cell-clusters.

Other methods to increase throughput can be achieved by increasing the flow rate and detector sensitivity (camera with high quantum efficiency). Special CCD and sCMOS cameras are better suited for imaging cytometry applications. These cameras can be wisely re-arranged to work in parallel, both spatially and temporally. Techniques such as time-delay and integration (TDI) in high-speed cameras have been shown to image objects along one axis at low light levels^45^. These specialized cameras allow row synchronization with the motion of moving cell images across the sensor array. Another efficient technique is multiple field-of-view imaging flow cytometry (MIFC) that enables high-throughput by projecting multiple FOVs onto the CMOS camera^46^ and by temporal coded excitation that controls the exposure time so as to reduces motion blur caused by flowing cells^47^.

Compared to the existing state-of-the-art flow based bioimaging systems, VFC enables live 3D volume visualization of flowing cells, high throughput interrogation (parameter estimation at approximately $800\,{\rm cells / min}$), and high-resolution imaging of sub-cellular organelles (mitochondria). For the VFC system, the lateral resolution is 3.14 µm at low flow-rates (50 nl/min) and the axial resolution is found to be about 3.75 µm. The relatively large lateral resolution is due to the refractive index mismatch (air-PDMS-cell medium). Apart from using long working-distance objectives with high NA, other ways to improve the lateral resolution is to match the refractive indices (PDMS-cell medium). This further minimizes undue reflection and optical aberrations. VFC has a cell throughput comparable to that of existing flow imaging-cytometers, indicating its suitability for analysing a large population of cells for intra- and inter-cellular investigations. Multi-channels microfluidic chip facilitates the interrogation of large cell populations in parallel at moderate flow rates without compromising high throughput. We employed VFC system to investigate the mitochondrial distribution in a large population of cancerous HeLa cells. The fact that organelles can be fluorescently-labeled adds value and expands its horizon beyond conventional flow cytometry. Recent studies include, fluorescence imaging by frequency division multiplexing^48,49^ and time-delay integration^50,51^, where fluorescence images have played key role in determining critical parameters. We envision that a low-cost implementation of the VFC system may be of immediate use in biomedical imaging.

The issue with conventional imaging flow cytometry is point-focussing PSF for interrogating flowing specimens. Such a system requires complex instrumentation involving hydrodynamic flow-focusing, thereby making it bulky and cumbersome. As a result, the system can interrogate one cells at any time-point. Such an arrangement leads to low throughput interrogation and cannot produce organelle-level information (organelle size, shape, and distribution). In contrast, VFC uses a sheet of light to illuminate the specimen and a multichannel microfluidic chip for the controlled flow of cells. Hence, VFC eliminates the need for hydrodynamic focussing and promotes simultaneous interrogation of multiple cells flowing through a single channel. This is a huge advantage considering the slow nature of sequential scanning used in conventional flow cytometry systems. Specifically, the VFC system is capable of accelerated cytometry by simultaneously imaging specimens in multiple channels (Y-type microfluidic-chip) in-parallel. In general, volume imaging is not feasible with the existing cytometry system due to the nature of system PSF (point-PSF). However, the proposed volume cytometry system is inherently designed to take cross-sectional images primarily due to sheet-shaped PSF and enables real-time volume reconstruction. Note that the convolution of individual 2D sectional images needs to be carried out before they can be stacked together to obtain volume-stack (see, Methods section and Supplementary 3). This necessitates the knowledge of system PSF. Point sources (fluorescent beads) are flown at varying flow-rates to determine the PSF-spread due to motion-blur. The PSF-distortion is found to vary linearly with the flow in the range 50–2000 nl/min (see, Fig. 3), limiting the number of recorded images obtained per cell somewhere between 4 and 8. Thereby flow-speed limits the information gathering capability of the VFC system.

Our findings further corroborate the organelle-level resolving capability of VFC system. In-principle, an orthogonal widefield detection ensures near diffraction-limited resolution, but we seldom reached this limit^52^. This is primarily due to the optical aberration (motion-blur) caused by flow in the microfluidic channels. Consequently, the resolution of VFC system is degraded by motion induced blur, and associated optical aberrations. Our results show that the system resolution worsens by a factor of 1.86 at large flow-rate (2000 nl/min). So the scaling factor is somewhere between 1.0 and 1.86, with the factor 1.0 for static specimen, and increases to $\eta _f =1.86$ at a flow-speed of 2000 nl/min. This gives a system resolution of, $(\lambda / 2 NA) \times \eta _f \approx 1.786$ µm at 2000 nl/min. However, the resolution is good enough to investigate intercellular organelle distribution in a cellular environment. Noting that the size of mitochondria in a cell varies somewhere between 1.0 and 6.5 µm (see, Fig. 7 and Supplementary 3), we can safely assume the resolution to be sufficient for studying mitochondrial distribution in the cell-cytoplasm. For the sake of comparison, we have compared reconstructed cell volumes with a confocal system as described in Supplementary 4.

Quantitative analysis shows that the probability of cell rolling is low, and the degree of cell’s rolling to be $< 2.76$ degrees for flow-rates $< 2500 \,\mathrm{nl}/\mathrm{min}$. The small rotation is predominantly due to laminar flow in the range 500–2500 nL/min. In addition, we noted that a short interaction time between the cell and light sheet during flow substantially reduces the probability of cell roll. However, rotation of specimens may need to be incorporated for non-laminar flow.

Imaging flow cytometers are known for interrogating a large number of specimens, producing statistics of critical features. High-speed interrogation introduces motion blur, resulting in poor axial resolution. However, there is a trade-off between throughput and resolution, as high-speed imaging requires short exposure to obtain images with minimal blur. Short exposure reduces blur at the cost of reduced fluorescence photons, while long exposure time increases photon collection at the expense of increased motion blur. In this regard, it is important that images of specimens are acquired using an exposure time that minimizes motion-blur while collecting as many fluorescence photons as possible. To address this limitation, VFC employs optimal exposure time, background subtraction, and image deconvolution using flow-variant PSF, all of which have shown marked improvement in image quality. Specifically, this has facilitated better estimation of biophysical parameters related to cell size, morphology, strain, and intra-cellular features.

The diversification of VFC lies in the use of other advanced light sheets such as the Bessel light sheet and the airy light-sheet^53–58^. These specialized light sheets offer a variety of advantages when compared to traditional Gaussian light sheets. The Bessel light-sheet brings in depth-penetration (due to non-diffracting nature) and self-reconstructing capability (reconstruction even after encountering several micro-obstacles). The beam facilitates illumination of target specimens at large penetration depths in inhomogeneous and scattering media containing several layers of microstructures and scattering micro-particles. On the other hand, airy light-sheet provides a wide field-of-view, propagation-invariant intensity profile and are known to exhibit self-healing. As a result, the airy beam light-sheet yields high contrast and resolution when compared to traditional Gaussian beam light sheet.

An important aspect to explore is label-free VFC imaging for real applications in human disease diagnosis. Methods such as chemical imaging flow cytometry are demonstrated by Suzuki et al.^59^ and Hiramatsu et al.^7^. The techniques use highly sensitive vibrational spectroscopy primarily based on stimulated Raman scattering. It demonstrates label-free single-cell analysis of cancer cells and the photosynthetic dynamics in H. lacustris cells. In another application, the bright-field images integrated with supervised machine learning have shown promise^60^. The technique is used for morphological feature extraction and found to be effective in cell cycle analysis for live mammalian cells. Label-free imaging is futuristic due to its ability to interrogate specimens in their native state and facilitate marker-free disease detection.

The access to organelle-level resolution coupled with healthy count (see, Fig. 5) and multi-parameter estimation (see, Fig. 7) makes VFC a unique system that combines the benefits of high-resolution microscopy, instant volume visualization, and high-throughput cytometry. Therefore, VCF system has the potential to emerge as the next-generation flow cytometric system for better disease prognosis in the broad field of biomedical engineering and imaging.

## Methods

### System design

The VFC imaging system has three major sub-systems: lightsheet illumination, multichannel microfluidic chip (specimen holder) and high-speed detection.

#### Lightsheet illumination

Simultaneous illumination of multiple microfluidic channels allow interrogation of a large population of cells. Accordingly, a large light sheet is generated in order to cross-section the entire array of micro-channels. A laser (532 nm Excel Laser, Quantum Lasers, UK) of wavelength 532 nm and beam-width 1.5 mm is used as the light source. The beam is expanded using a beam-expander (consists of two biconvex lens of focal-lengths 25 mm and 125 mm procured from Thorlans, USA ) by a factor 5*X*-times. This is essential to just over-fill the back-aperture of cylindrical lens ($f=150$ mm, Edmund Optics, Singapore) to utilize its full NA. The expanded beam is 1D-focused by the cylindrical lens to form a horizontal light-sheet. An objective lens (Olympus 10X, 0.30 NA) is placed at the focus of cylindrical lens to generate diffraction-limited light sheet. The resultant light sheet served as the illumination PSF of VFC system.

#### Microfluidic chip based sample flow system

Cells were flown through the Y-shapped microfluidic channel array as shown in Fig. 1 and Fig. S1 (see, Supplementary 1). The microfluidic chip is fixed to a home-built chip holder and placed on a XYZ-translator for precise position with respect to light sheet. This enabled optical sectioning of the entire channel array (an array of 4 channels of size 100 µm^2^). The channel inlet is linked to sample reservoir containing the cells whereas, the outlet is connected to a flow-pump. The pump is operated in a suction-mode and the operations are controlled by computer based interface software. Both the specimens, fluorescent bead (FluoSpheres F8803, Invitrogen) and HeLa cells (labelled with Mitotracker Orange CMTMRos, ThermoFisher) were flown through the channel-array and imaged in-parallel.

The proposed VFC technique uses oblique illumination coupled with orthogonal detection geometry. While the configuration is similar to that of classical SPIM, the presence of a PDMS-based microfluidic chip introduces optical aberration primarily due to refractive index mismatch as the light pass through air-PDMS-sample interfaces. Specifically, the mismatch between the specimen flow device (PDMS) and the sample buffer (cell medium) introduces aberrations, thereby degrading the image resolution. To minimize the effect, we designed the microfluidic channel so as to avoid undue refraction, especially at the chip edges. Accordingly, the fabricated open channels are first realized and then sealed with a microscope-grade refractive-index matching coverslip. The sealing was carried using an oxygen plasma machine where oxygen plasma treatment of both the glass substrate and PDMS’s surfaces are carried out before contacting them with each other immediately after activation. This produces a strong and irreversible seal. The geometrical issue associated with PDMS microfluidic device can be effectively addressed by constructing the channel from a material (such as Teflon, RI=1.35–1.38) with the same refractive index as the cell flow medium (RI= 1.33–1.38). This could resolve many issues arising out of refractive index mismatch. Future improvements require that the number of material interfaces is reduced within the light-path to avoid aberration occurring due to reflection and refraction. Another critical issue is associated with the preparation and cutting of PDMS microfluidic devices that may introduce irregular interfaces (air-PDMS). An additional step to flatten the surfaces could go a long way. All these factors strongly affect image quality and, ultimately the 3D volume.

It may be noted that the angle between the microfluidic chip and light-sheet illumination is critical. The design geometry requires the specimen flow chip to be placed at $45^{\circ }$ to the illumination system, and the detection is carried out at an oblique angle of $45^{\circ }$ to collect the light sheet images. The detection scheme is similar to that used in open-top light-sheet (OTLS) microscopy^61,62^. Oblique light sheet images are recorded and stored initially in a rectangular data cube. Subsequently, the data is sheared to represent the geometry of the reconstructed volume.

#### Fast detection

 The mitochondria in HeLa cell was labelled and the emission peaks at 576  nm. The fluorescence from specimen (HeLa cells) is collected by the detection objective (Meiji, 20X, $0.4\,NA$). Subsequently, the light is filtered by a set of filters (notch filter (ZET532nf purchased from Chroma) to remove the illumination 532 nm light and a long-pass filter (purchased from Thorlabs) to filter-out the background to retain fluorescence. The filtered light is then focused to the camera chip (pixel size ~ 5.5 µm × 5.5 µm) by a tube lens ($f=125$ mm). The detector is a superfast CCD camera (GZL-CL-22C5M-C, Point gray, USA) with a maximum frame rate of $2.3K \,{\rm frames/s}$ and have a quantum efficiency of 0.56. The images were collected and sent to the computer for further processing.

### System point spread function

To determine the system PSF, fluorescent beads (size = 1 µm) were flown through the microfluidic channels at varying flow-rates. The beads recorded by the camera are appropriately modelled as point source and characterized.

Beads flowing through the channel are conveniently modelled as 2D Gaussian,

1 $$\begin{aligned} f(y,z) = G_0 \,e^{-[(y-y_0)^2 / 2\sigma _y^2 + (z-z_0)^2 / 2\sigma _z^2 ]} \end{aligned}$$

where, $G_0$ is a constant. Here, $z_0$ and ($\sigma _x , \,\sigma _y$) are respectively the mean and standard deviations.

The diameter of bead is approximately given by its full-width at half-maxima (FWHM) i.e,

2 $$\begin{aligned} D \approx FWHM \approx 2\sqrt{2ln 2} \,\sigma \end{aligned}$$

During flow, the beads undergo motion-blur. As a result, the beads appear elongated in the recorded image. Considering a flow-rate of *Q* through the channel of cross-section *A*, and a camera exposure of $t_{exp}$, the elongation of a point (along the direction of flow) is given by,

3 $$\begin{aligned} \Delta z = v\,t_{exp} =\frac{Q}{A}\,t_{exp} \end{aligned}$$

where $v=Q/A$ is the average velocity of fluid flowing (at a flow-rate, *Q*) through the channel of cross-section *A*.

Due to flow, the detection PSF appear elongated along the flow direction (z-axis). So the changes in $\sigma _z$ in the recorded image can be expressed as,

4 $$\begin{aligned} \sigma _z = \frac{1}{2\sqrt{ 2 ln 2 }} FWHM_{z} = \frac{(D_z +\Delta z)}{2\sqrt{ 2 ln 2 }} = \frac{(D_z + v\,t_{exp})}{ 2\sqrt{ 2 ln 2 } } \end{aligned}$$

where, $D_z$ is the diameter in static condition, and $FWHM_z$ is the full-width at half-maximum of PSF along z-axis.

The standard deviation for the beads along y- and z- axes are given by,

5 $$\begin{aligned} \left\{ \begin{array}{ll} \sigma _y = \sigma _{y0} = D_y/2\sqrt{2ln 2} \\ \\ \sigma _{z} = \left[ \sigma _{z0} +\frac{v \,t_{exp}}{{2\sqrt{2ln 2}} } \right] = \frac{1}{2\sqrt{2ln 2}} \,\left[ D_z +\frac{Q\,t_{exp}}{A} \right] \end{array}\right. \end{aligned}$$

where, $\sigma _{y0}$ and $\sigma _{z0}$ are respectively the standard deviation along *y* and *z* axis at zero velocity ($v=0$).

So, the beads flowing at a flow-rate *Q* can be approximated by a bivariate Gaussian PSF given by,

6 $$\begin{aligned} G_Q = G_0 \,\exp { \left\{ -\left[ \frac{(y-y_0)^2 }{ 2\sigma _y^2} + \frac{(z-z_0)^2 }{ 2\sigma _{z}^{2}} \right] \right\} } \end{aligned}$$

where, $\sigma _y$ and $\sigma _z$ are as given by Eq. (5).

Experimentally, the images of bead samples (YZ-plane) are recorded. A bivariate-Gaussian function is fit to calculate the flow-induced shifts (along *z*-axis) with respect to beads at $v=0$. With the calculated variances ($\sigma _y$ and $\sigma _z$), a new 2D Gaussian is generated, which is then used as the system PSF. The process is carried out for all flow-rates. The corresponding system PSF at different flow-rates is the used for reconstruction sectional images (see, details in Supplementary 3). For our case, we have taken fluorescent bead of size 1 µm for which the emission occurs at $\lambda _{em} = 575$  nm, and recording is carried out for all flow-rates.

### Image reconstruction using flow-variant PSF

For VFC, the recorded image ‘g’ of the object (cell) ‘o’ flowing through the channel can be modelled as,

7 $$\begin{aligned} g(y,z) = h(y,z) \otimes o(y,z) \end{aligned}$$

where, *h* is the PSF of the dynamic flow system, and $\otimes$ denotes the convolution operator.

In the Fourier domain, the above equation can be expressed as,

8 $$\begin{aligned} G = H \times O \end{aligned}$$

where, *G*, *H*, *O* are respectively the Fourier transform of the recorded image ‘*g*’, point spread function ‘*h*’ and object function ‘*o*’.

We seek the object function ‘*o*’ from the recorded image. The function can be retrieved from the above equation by inversion. Computationally, this is accomplished by inverting the function in Fourier domain followed by inverse Fourier transform i.e,

9 $$\begin{aligned} o(y,z) = F^{-1} \{ G / H \} \end{aligned}$$

The above expression is a well-known inverse problem, with the exception that here ‘h’ is a flow-variant PSF. The entire process is commonly known as deconvolution.

We have used HeLa cells (of size, 15–25 µm), and the mitochondria is labelled using mitotracker orange dye following the process described in sample preparation section. The cells were flown at different flow-rates and the images were recorded at a video-rate of $38 \,\mathrm{Hz}$. The ergonomic design of our system allowed collection of 4–6 sectional images as the cells pass through the light sheet. The 2D images were deconvolved using flow-variant PSF and images were reconstructed. Subsequently, 2D sectional images were stacked together to reconstruct the cell volume (see, Supplementary 3 and 5).

### Microfluidic chip fabrication

Using Clewin 4, channel features are designed on 4-inch silicon disc. Negative mask is printed from Clewin 4 .gbr file. Master mold fabrication is done in clean room facilities at Nanoscience Facility, Indian Institute of Science, Bangalore, India. Subsequently, Y-type microfluidic chips were fabricated using standard protocol. Silicon elastomer and curing reagent are mixed thoroughly in the ratio of 10:1. A net mixture amount of 33gm is dicicated with vacuum pump for 20 min until air bubbles are removed. Decicated mixture is gently poured on the top of master mold and is cured in hot oven at $60\,^{\circ }$C for 3 h. Cured PDMS is peeled off from master mold and useful region are extracted from it by cutting. Thus, replica of the micro-channels on the PDMS blocks are obtained. Inlet and outlets are punched with 1.0 mm diameter PDMS puncher and cleaned with isopropanol and acetone. Washed PDMS with microchannel channel and bonding glass (0.15 mm thickness) are plasma cleaned for 5 min. Soon after plasma cleaning is completed, PDMS is placed on the top of coverslips followed by baking on hotplate for 5 min at $90\,^{\circ }$C. The description of master mold and fabricated Y-type microfluidic chip can be found in Supplementary 1. Using microfluidic Teflon tubing (inner diameter of 0.5 mm) reservoir is connected to inlet and outlet is joined to the flow pump (New Era Flow Pump, Model No: NE-1002X). Microfluidic chips were ensured leakage-free by flowing distilled water while the features of micro-channels are obtained using low concentration TRITC solution ($Ex/Em = 557/576$ nm) before carrying out actual imaging with beads and HeLa cells.

### Flow pump and data acquisition

The 2D sectional images of HeLa cells were recorded by the CCD/sCMOS camera. Two major systems were synchronized to record the data, (1) flow control system and (2) Lab-view Image Acquisition. The flow is controlled using a flow-pump operated in a withdrawl mode, and at flow-rates ranging from 500 to 2000 nl /min (for HeLa cells).

Needle of flow-pump (BD,1ml conventional syringe of diameter 4.80mm) is attached to outlet tube of the microfluidic device to withdraw the cell embedded solution from the reservoir through the micro-channels. Volumetric flow rate (in nanolitre cube) is the set point on pump. Dispense module is activated for withdrawal-mode of the pump. The pump was operated from 500 to about 2000 nl/min for data acquisition.

Raw image data are recorded using both CCD camera (Point gray camera, Model:GZL-CL-22C5M-C, Point gray, USA) and sCMOS (Zyla 4.2, Andor Tech., UK). For accurate and fast data collection the camera is interfaced with NI PCI 1433 card (frame-graber device) and is programmed using LabVIEW-12. Set point on the program are exposure-time and camera resolution. Optimum value of exposure time is decided by the thickness of light sheet and flow-rate (see, Eq. (3) ). The camera (pixel size = 5.5 µm) is configured to take data at full-frame of $2048 \times 1088$. Recorded data / images are saved in $*.bmp$ format.

### Sample preparation

#### Fluorescent beads

Beads were used for both calibration and as a test sample. $1 \mu l$ of Invitrogen FluoSpheres Carboxylate-Modified Microspheres, $1.0 \upmu \mathrm{m}$ diameter (Nile Red fluorescent, $Ex: 535\,\mathrm{nm} / Em: 575$ nm) (Invitrogen, USA)is suspended in $1 \,ml$ of distilled water. Through mixing is done by pipetting and the mixture is loaded to the sample reservoir of VFC system for counting, imaging and parameter estimation.

#### Cell line and maintenance

HeLa cells (human cervical carcinoma cell line) obtained from our collaborator Dr. Upendra Nongthomba (Biological Sciences, Indian Institute of Science, Bangalore, India) were used for the experiment. The HeLa cells were cultured and maintained in incubator in complete Dulbecco’s modified minimal Eagle’s medium (DMEM) (Gibco, Thermo Fisher Scientific) supplemented with $10\%$ FBS( Gibco, Thermo Fisher Scientific) and $1\%$ penicillin –streptomycin solution (Gibco, Thermo Fisher Scientific) at $37\,^{\circ }$C and $5\% \,CO2$ (CO2-incubator, Thermo Scientific). After 2 passage, the cells were prepared for experiment. Hemocytometer is used to count cells after every passage and approximately 100,000 cell count was maintained. The cells were passaged in every 2–3 days to maintain healthy cell-lines.

#### Mitochondrial labelling using mitotracker orange

Cells were seeded ($10^5$ cells) for 24h in complete DMEM. To evaluate optimum mitotracker orange concentration needed with respect to the cell density, staining efficiency and quantum yield in imaging flow cytometry, 6 different working concentration of mitotracker orange (100, 125, 150, 175, 200, 225 nM) in DMEM were tested. Our study revealed, 175 nM mitotracker orange concentration gives optimal mitocondrial staining. For studies presented in this study, the HeLa cells (at $70\%$ confluence) were treated with 175 nM mitotraker orange in DMEM for 15 mins in incubator. Further, the cells were trypsinized and washes three times with phosphated buffered saline (PBS) to remove trypsine and cell debris by centrifugation (300xg for 5 mins). Subsequently, the cells were resuspended in PBS and prepared for experiments. The cells were loaded in the sample reservoir and flown through microfluidic chip for volume imaging cytometry.

## Supplementary Information

Supplementary Information 1.

Supplementary Information 2.

Supplementary Information 3.

Supplementary Information 4.
