# Supplementary material for: Light sheet based volume flow cytometry (VFC) for rapid volume reconstruction and parameter estimation on the go
Source: Sci Rep. 2022 Jan 7;12:78. doi: 10.1038/s41598-021-03902-8 (PMC8741756; doi:10.1038/s41598-021-03902-8)
Supplement: Supplementary file 1 — Supplementary Information 1. [file 41598_2021_3902_MOESM1_ESM.pdf]

Supplementary Information | Light Sheet Based Volume Flow Cytometry (VFC) for Rapid Volume Reconstruction and Parameter Estimation On The Go

Authors : Prashant Kumar, Prakash Joshi, Jigmi Basumatary and Partha Pratim Mondal

Supplementary 1: Microfluidic Chip Fabrication and Specimen Flow Device

Supplementary 2: Protocol for Cell Counting, Volume Imaging and Bioclinical Parameter Estimation

Supplementary 3: Deconvolution using Flow-Variant PSF and Volume Reconstruction

Supplementary 4: Comparison of Volume Flow Cytometry and Confocal System

Supplementary 5: Morphology of Reconstructed Mitochondrial Network in HeLa cells

Supplementary Videos:

Supplementary Video 1: Raw data of fluorescent beads at varying flow rates (500 nl /min – 2000 nl / min).

Supplementary Video 2: Recorded raw data of HeLa cells flowing through all the 4 microfluidic channels (flow rate of 2000 nl / min).

Supplementary Video 3: Slow-motion of a typical HeLa cell passing through the light sheet in one of the channels.

Supplementary 1: Microfluidic Chip Fabrication and Specimen Flow Device

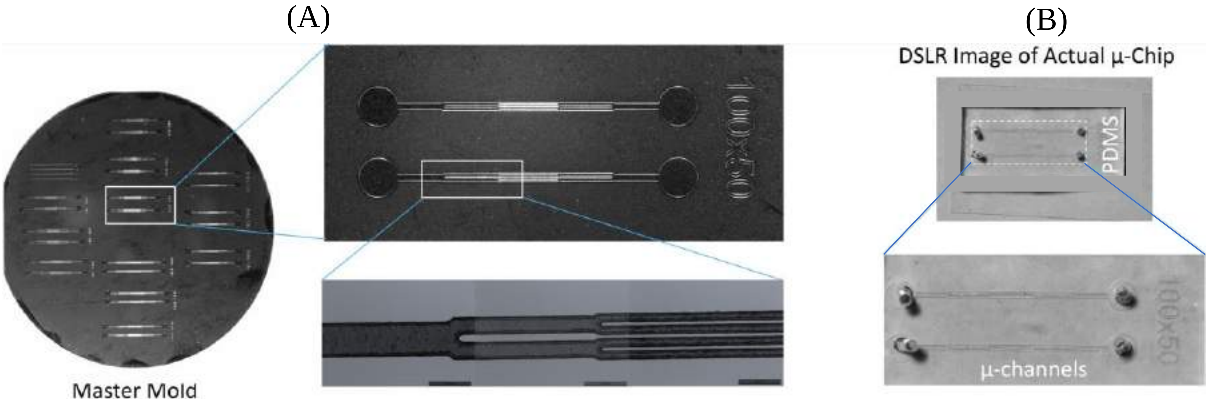

Fig. S1: Microfluidic chip fabrication. (A) The master mold with a micro-channel array of various sizes is fabricated on a Silicon wafer with SU-8 material as the photosynthesizer. (B) DSLR image of actual m-chip using PDMS. Y-type microfluidic channels of size, 100 × 50 microns (size=100 µm with an inter-channel separation of 50 µm) can be seen.

Microfluidic channels used in the VFC system were fabricated using a master-mold as shown in Fig. S1. The negative photoresist SU-8 2100 was patterned onto a Silicon wafer to get a master with features of dimension 100 mm ´ 100 mm (W ´ H) using a standard photolithographic technique. The mixture of polydimethylsiloxane (PDMS) and its curing agent (ratio of 10:1) from Dow Corning's Sylgard 184 elastomer was poured on the master to get the required pattern in PDMS (Friend and Yeo, 2010) [1]. Finally, the patterned PDMS was bonded to microscope coverslip by oxygen plasma treatment for 3 minutes.

The fabricated microfluidic channels are shown in Fig. S1(B). Post fabrication, holes of the dimension of external tubes were punched at both the ends of the Y-type channel-array (containing four connected channels). A conical reservoir is connected at one end, and the other end connects to the suction-pump. The flow in the channel-array is controlled in the range of 50 to a few thousand nano-lit / min using an external pump (operated in withdrawal mode).

Supplementary 2: Protocol for Counting, Volume Imaging, and Biophysical Parameter Estimation

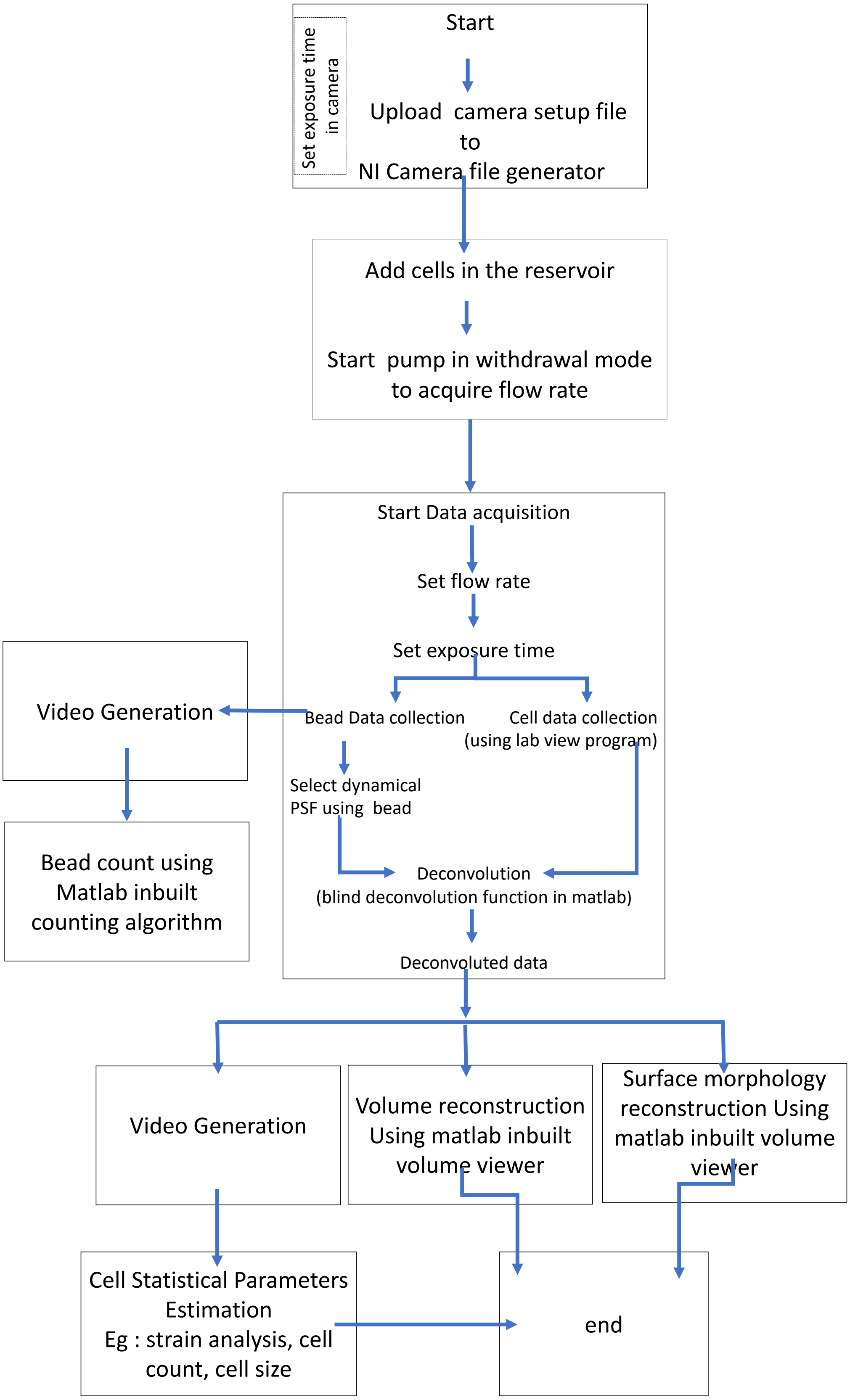

Fig. S2. Basic flow-chart depicting the general protocol for operating semi-automated volume flow cytometry system.

VFC system is a semi-automated cytometry system that requires manual addition of cell sample in the reservoir and allocation of detection parameters (such as exposure time and background reduction). A detailed protocol for operating VFC imaging cytometry system is shown in the flow-chart S3. The data collection begins by loading the reservoir with cell samples and fixing the imaging parameters (frame-rate, flow-rate, and PSF characteristics).

Labview platform is used for synchronizing laser, sCMOS camera, and flow control through microfluidic channels (housed in PDMS-based microfluidic chips). Data acquisition is carried out for 10 minutes duration at each flow-rate. The fluorescent beads with known bead-size are flown at varying flow-rates, and the calibration curve is generated. This gives the estimation of flow-variant PSF. Subsequently, the cell samples are flown at different flow-rates, and the sectional 2D images are collected. The 2D images are then deconvolved using the calibrated flow-variant PSF. At this stage, the data is ready for counting, volume visualization, rendering, and bioclinical parameter estimation.

MATLAB programming language is used for volume visualization and rendering. From these volume data, the parameters are estimated, and the relative plots are generated (see, Fig. 7 in the main manuscript). Separate Matlab scripts are developed for counting.

Supplementary 3: Deconvolution using Flow-Variant PSF and Volume Reconstruction

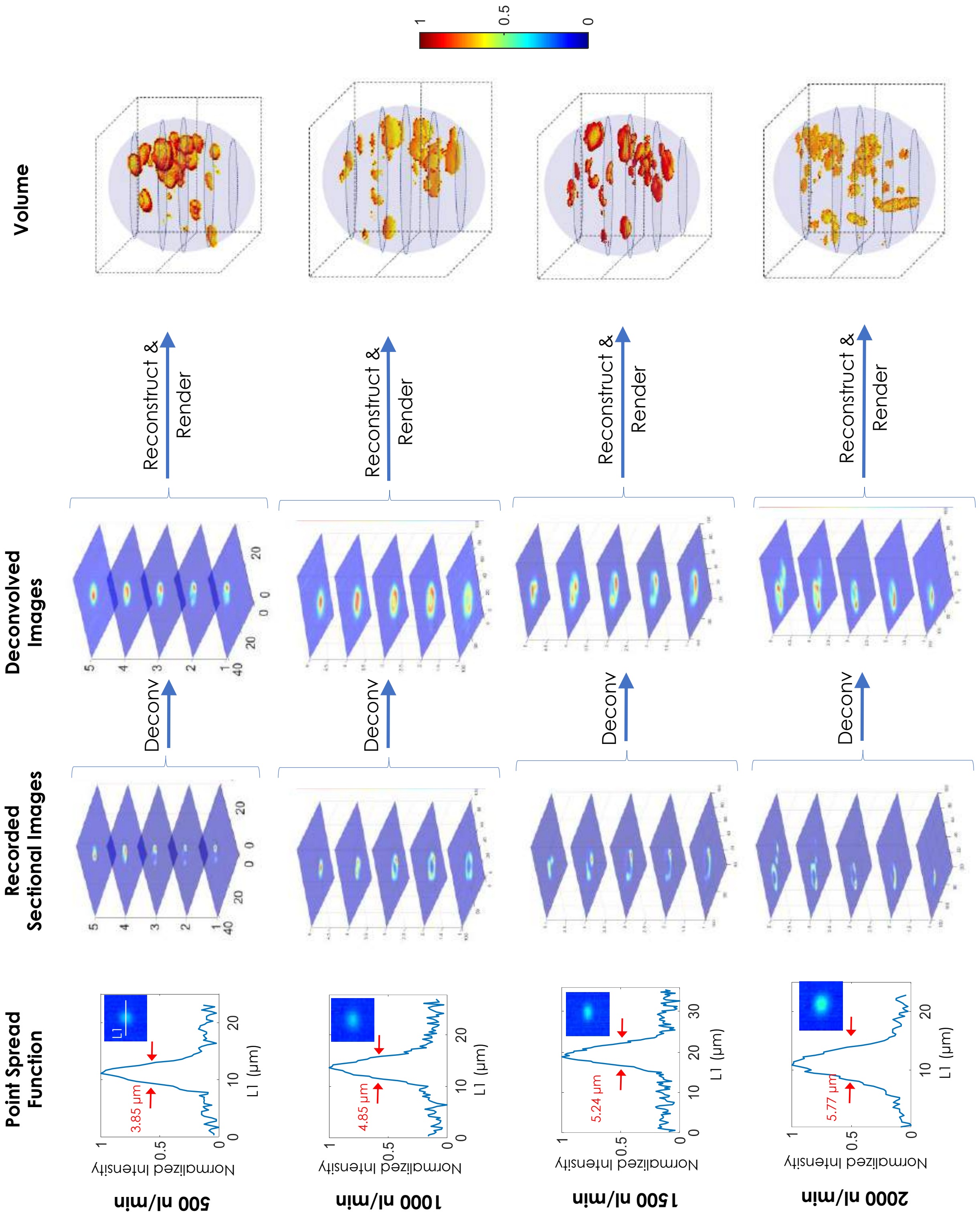

Fig. S4. Deconvolution of recorded images of HeLa cells using flow-variant PSF at flow-rates, varying from 500 nL/min to 2000 nL/min.

Light sheet imaging flow cytometry system undergoes convolution, and so deconvolution plays a crucial role. In addition, the recorded images experience motion-blur due to samples moving through the microfluidic channels. So, traditional deconvolution techniques cannot be applied to flow cytometry images, and hence a new approach is needed. The PSF needs to consider motion-blur in the image reconstruction method to deconvolve the recorded data accurately. So, we have used fluorescent beads as point sources and flown at varying flow-rates. From the recorded images the motion induced blur / deformation is calculated and flow-variant PSF is determined that can be used for deconvolving recorded images.

Recorded sectional images are then used to reconstruct the morphology of mitochondrial network. For volume reconstruction and rendering, the deconvolved images are stacked together to obtain the volume images as shown in Fig. S4. The distribution of mitochondria is visible in the associated 3D volume. From volume-stack, morphological changes are determined using developed Matlab scripts that provide details of organelle distribution (see, Fig. 6 in the main manuscript). VFC volume cytometry may become a potential clinical tool to identify sub-cellular changes in cells at high throughput.

.

Supplementary 4: Comparison of Volume Flow Cytometry and Confocal System

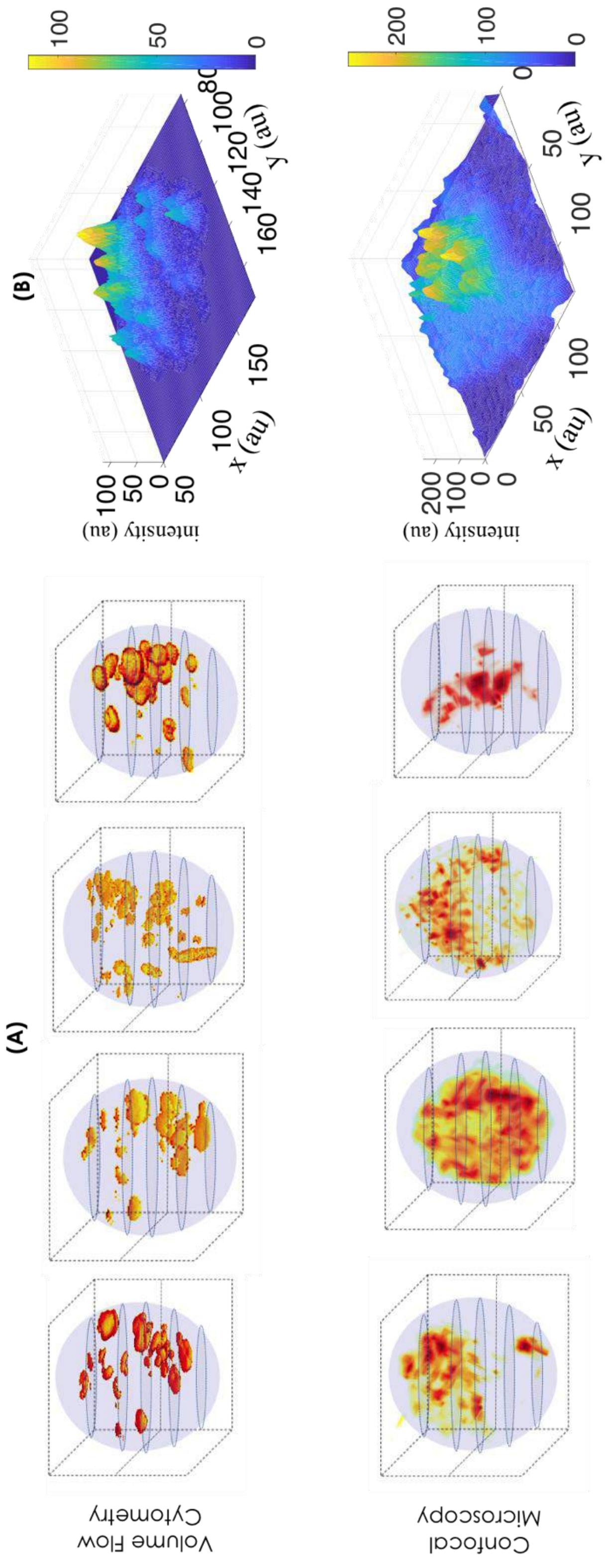

Fig. S5. (A) Comparison of flow-based VFC with the state-of-the-art confocal scanning fluorescence microscopy. (B) 3D surface plot of a typical HeLa cell that shows visual comparison of flow-based VFC and state-of-the-art confocal system.

To enable quality reconstruction, we have compared VFC images with state-of-the-art confocal images. Although flow-based VFC and static confocal system are not comparable, the study gives a visual comparison of system resolution. Images stacks of some of the HeLa cells are obtained from both the imaging modalities, as shown in Fig. S6A. To exemplify the details, we used 3D surface plots as shown in Fig. S6B. One can immediately recognize better resolved finer details in the confocal volume than relatively broad features in the VFC system. This indicates the absence of high frequencies in the VFC reconstructed volumes. The dynamic nature of VFC system is mainly responsible for relatively poor resolution. In addition, pin-hole-based detection in confocal outperforms orthogonal widefield detection in VFC. Because the proposed technique is a flow-based high-throughput imaging technique, the organelle-level resolution is quite commendable for extensive population screening.

Supplementary 5: Morphology of Reconstructed Mitochondrial Network in HeLa cells

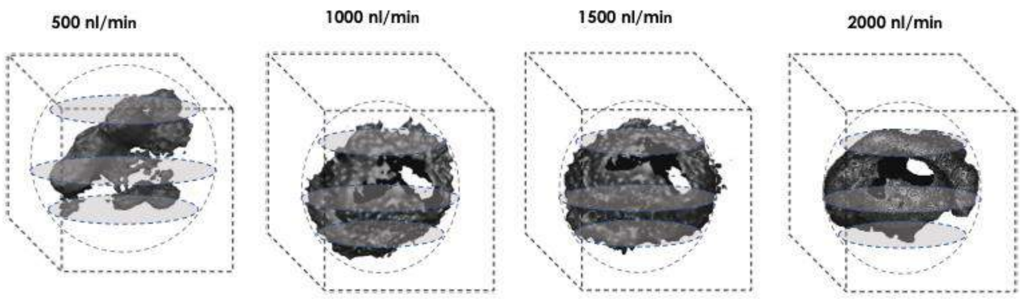

Fig. S5-1. Reconstructed 3D morphology of mitochondrial network in HeLa cells at varying flow-rates (500 nL/min to 2000 nL/min.)

The distribution of mitochondria and the morphology of mitochondrial network strongly relates to cell physiology. Here, we study the morphology of mitochondrial network in normal HeLa cells. Specifically, 3D reconstruction gives a better visualization of mitochondrial network. Fig. S5-1 shows the reconstructed volume of network in normal HeLa cell. The 3D cell volumes are shown at flow rates, 500 nl/min - 2000nl/min. The volumes are constructed from sectional images recorded during flow using developed MATLAB scripts. The mitochondrial network for HeLa cells seems to be well-connected, however the details are missing at large flow-rates.

An associated metric to measure information content for reconstructed cell volumes, we employ Shannon's entropy [2] [3]. By definition, Entropy is a measure of image/volume information content, which is interpreted as the average uncertainty of information source (here, cell). It is frequently used for the quantitative analysis and evaluation of image/volume details [4] [5]. Entropy for a volume that consists of q images, each with intensity / grey-level i is defined as,

$$E = -\sum_{q}\sum_{i} P_i \, log_2(P_i)$$

where P_i represents the histogram count for the grey-level i.

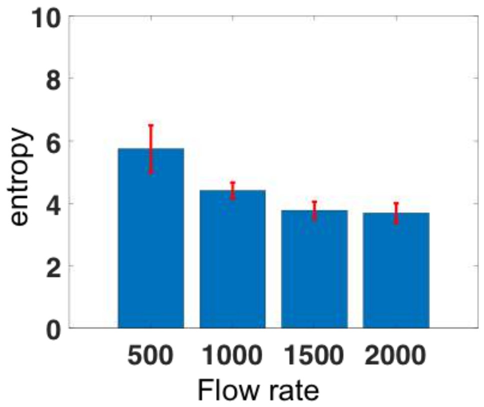

Fig. S5-2. Entropy (information content) of the reconstructed volumes of HeLa cells flowing through the microfluidic channels at varying flow-rates (500 μL to 2000 μL).

Information content associated with the mitochondrial network in the reconstructed volumes of HeLa cells are shown in Fig. S5-1. A decrease in entropy suggests loss of information at large flow-rates, indicating poor reconstruction of mitochondrial network. This is due to motion induced blur that reduce the high-frequency content in the reconstructed volumes.

Overall, the study suggests that the proposed VFC may serve as an alternate technique to screen a large population of Hela cells with organelle-level resolution. In the future, VFC may initiate studies leading to organelle-organelle interaction, changes, and their dynamics on the go.

References:

1. J. Friend, L. Yeo, Fabrication of microfluidic devices using polydimethyl-siloxane. Biomicrofluidics 4, 026502 (2010).
2. Du-Yih Tsai, Yongbum Lee, and Eri Matsuyama, Information Entropy Measure for Evaluation of Image Quality, J Digit Imaging 21, 338–347 (2008).
3. J. Lin, Divergence measures based on the Shannon entropy, IEEE Tran. Info. Theory, 37, 1 (2006).
4. F. De Giorgi, L. Lartigue and F. Ichas, Electrical coupling and plasticity of the mitochondrial network. Cell Calcium 28: 365–370 (2000).
5. V. P. Skulachev, Mitochondrial filaments and clusters as intracellular power-transmitting cables. Trends Biochem. Sci. 26: 23–29 (2001).
